# Supplementary material for: Persistent Moderate-to-Weak Mediterranean Diet Adherence and Low Scoring for Plant-Based Foods across Several Southern European Countries: Are We Overlooking the Mediterranean Diet Recommendations?
Source: Nutrients. 2021 Apr 23;13(5):1432. doi: 10.3390/nu13051432 (PMC8145023; doi:10.3390/nu13051432)
Supplement: Supplementary file 1 [file nutrients-13-01432-s001.zip › Supplementary Figure S1.pptx]

## Slide 1
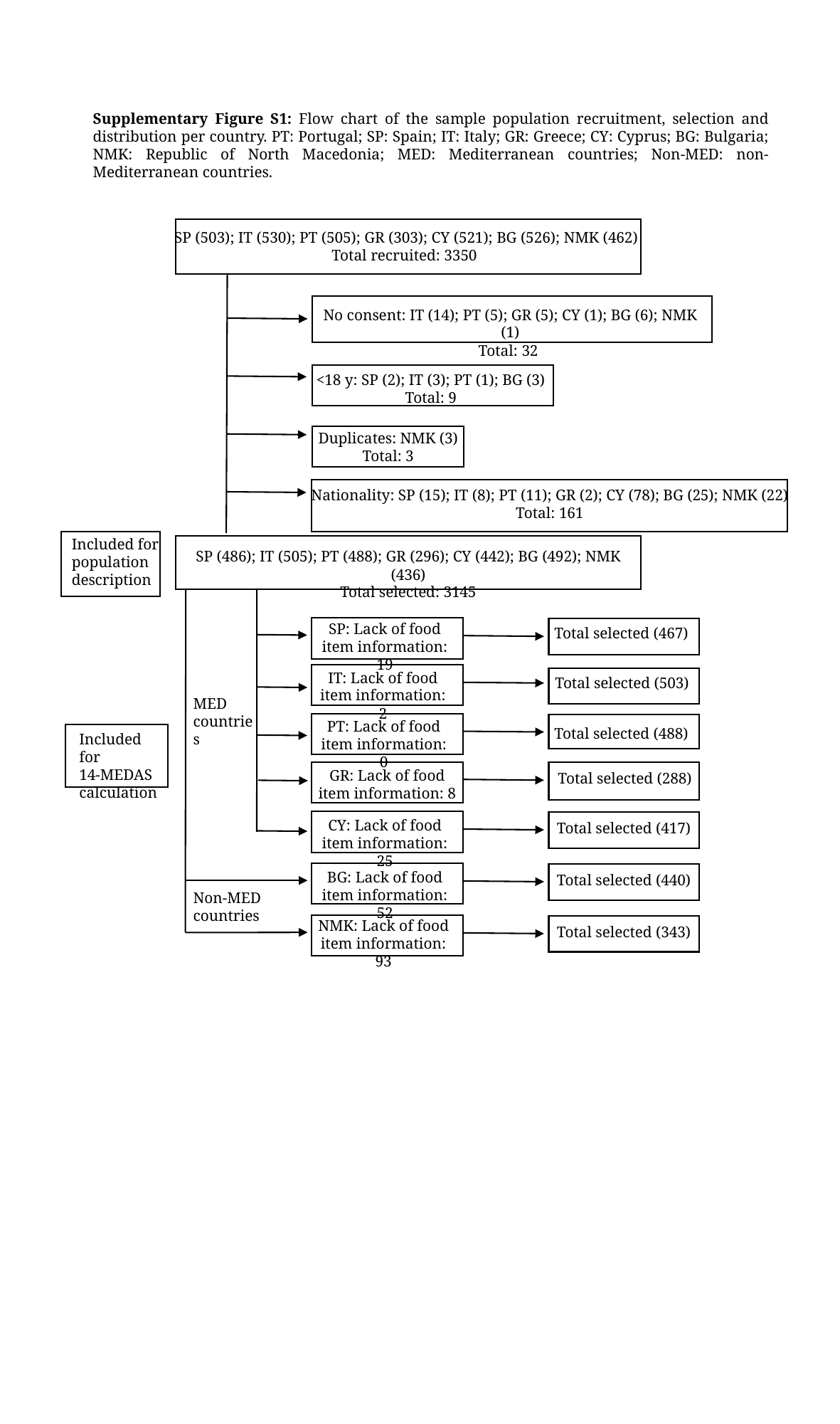

Supplementary Figure S1: Flow chart of the sample population recruitment, selection and distribution per country. PT: Portugal; SP: Spain; IT: Italy; GR: Greece; CY: Cyprus; BG: Bulgaria; NMK: Republic of North Macedonia; MED: Mediterranean countries; Non-MED: non-Mediterranean countries.
SP (503); IT (530); PT (505); GR (303); CY (521); BG (526); NMK (462)
Total recruited: 3350
No consent: IT (14); PT (5); GR (5); CY (1); BG (6); NMK (1)
Total: 32
<18 y: SP (2); IT (3); PT (1); BG (3)
Total: 9
Duplicates: NMK (3)
Total: 3
SP (486); IT (505); PT (488); GR (296); CY (442); BG (492); NMK (436)
Total selected: 3145
SP: Lack of food item information: 19
PT: Lack of food item information: 0
Total selected (467)
IT: Lack of food item information: 2
 Total selected (503)
Total selected (488)
Included for
14-MEDAS calculation
GR: Lack of food item information: 8
Total selected (288)
CY: Lack of food item information: 25
Total selected (417)
BG: Lack of food item information: 52
Total selected (440)
NMK: Lack of food item information: 93
Total selected (343)
Nationality: SP (15); IT (8); PT (11); GR (2); CY (78); BG (25); NMK (22)
Total: 161
Included for population description
MED
countries
Non-MED
countries
