# Supplementary material for: Persistent Moderate-to-Weak Mediterranean Diet Adherence and Low Scoring for Plant-Based Foods across Several Southern European Countries: Are We Overlooking the Mediterranean Diet Recommendations?
Source: Nutrients. 2021 Apr 23;13(5):1432. doi: 10.3390/nu13051432 (PMC8145023; doi:10.3390/nu13051432)
Supplement: Supplementary file 1 [file nutrients-13-01432-s001.zip › Supplementary Figure S2.pptx]

## Slide 1
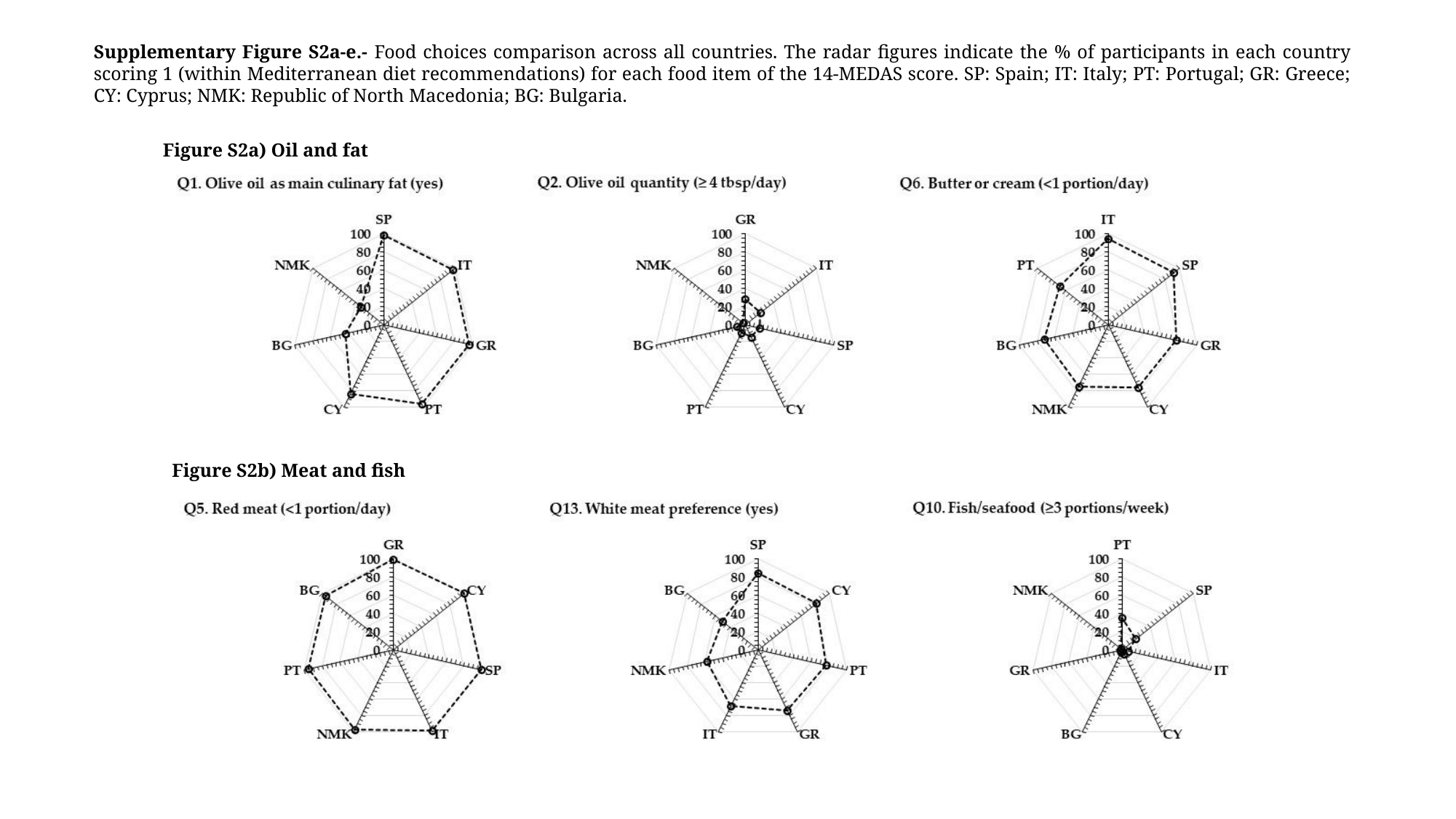

Supplementary Figure S2a-e.- Food choices comparison across all countries. The radar figures indicate the % of participants in each country scoring 1 (within Mediterranean diet recommendations) for each food item of the 14-MEDAS score. SP: Spain; IT: Italy; PT: Portugal; GR: Greece; CY: Cyprus; NMK: Republic of North Macedonia; BG: Bulgaria.
Figure S2a) Oil and fat
Figure S2b) Meat and fish

## Slide 2
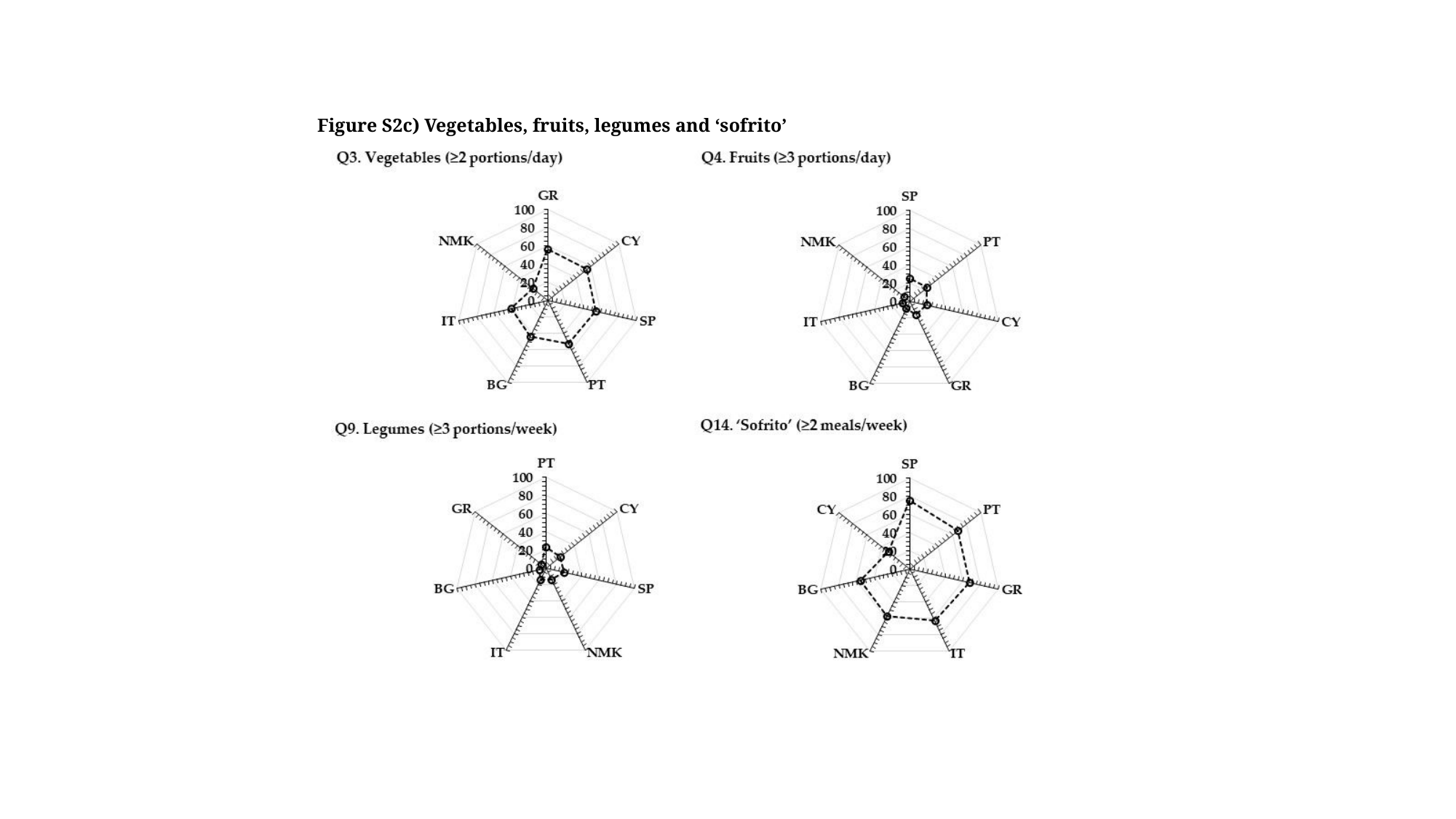

Figure S2c) Vegetables, fruits, legumes and ‘sofrito’

## Slide 3
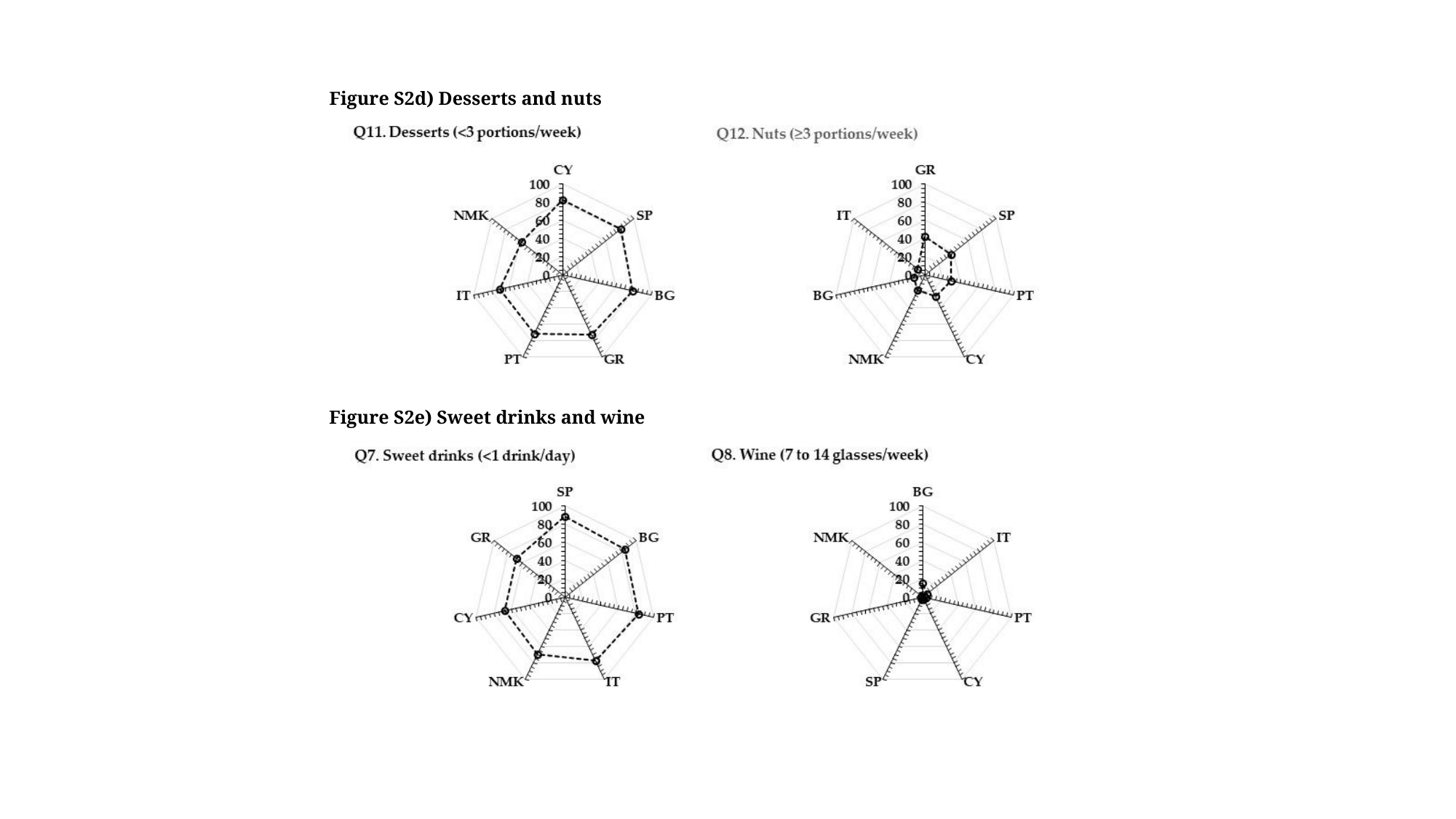

Figure S2d) Desserts and nuts
Figure S2e) Sweet drinks and wine
