# Supplementary material for: Persistent Moderate-to-Weak Mediterranean Diet Adherence and Low Scoring for Plant-Based Foods across Several Southern European Countries: Are We Overlooking the Mediterranean Diet Recommendations?
Source: Nutrients. 2021 Apr 23;13(5):1432. doi: 10.3390/nu13051432 (PMC8145023; doi:10.3390/nu13051432)
Supplement: Supplementary file 1 [file nutrients-13-01432-s001.zip › Supplementary Figure S3.pptx]

## Slide 1
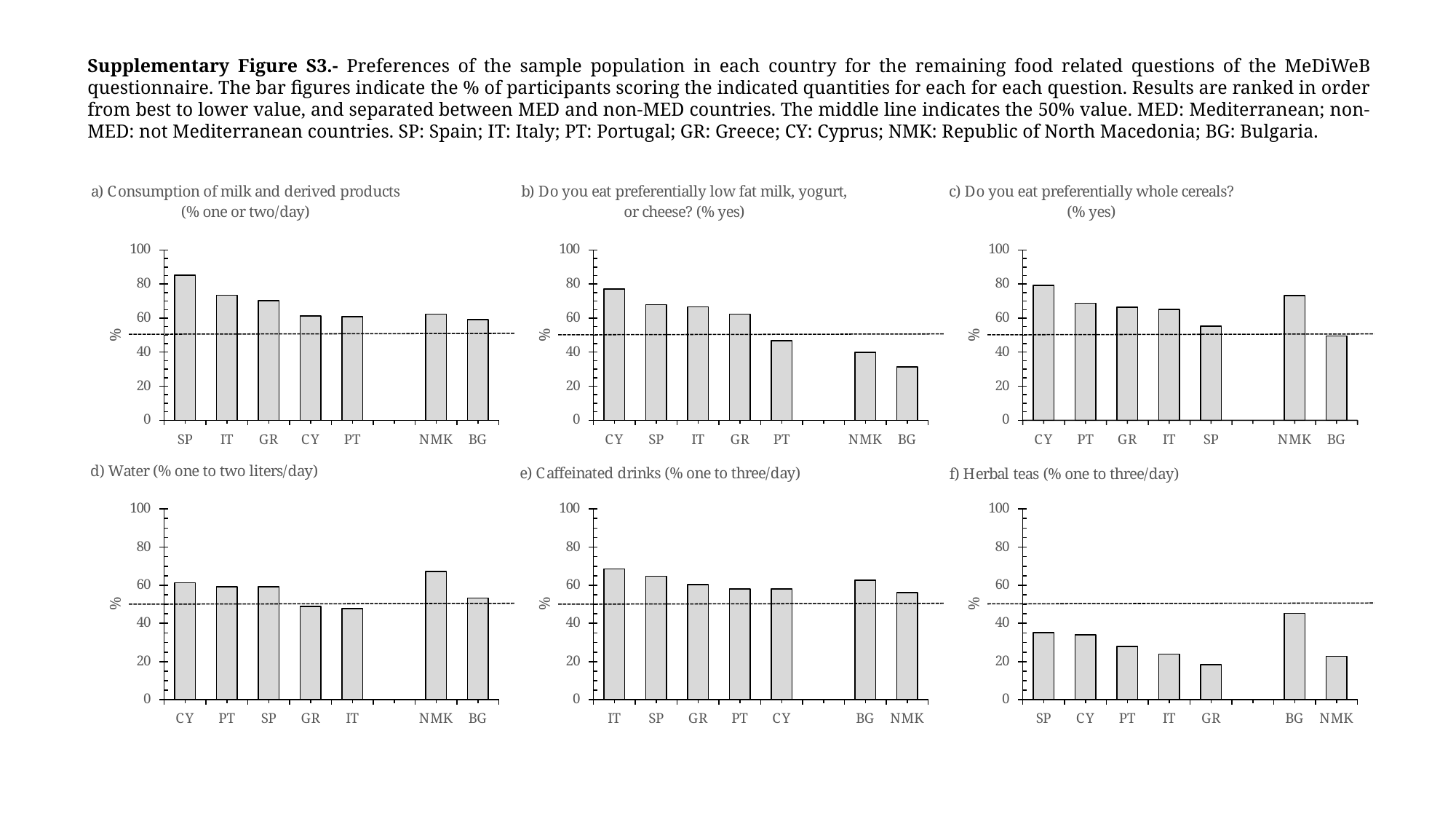

Supplementary Figure S3.- Preferences of the sample population in each country for the remaining food related questions of the MeDiWeB questionnaire. The bar figures indicate the % of participants scoring the indicated quantities for each for each question. Results are ranked in order from best to lower value, and separated between MED and non-MED countries. The middle line indicates the 50% value. MED: Mediterranean; non-MED: not Mediterranean countries. SP: Spain; IT: Italy; PT: Portugal; GR: Greece; CY: Cyprus; NMK: Republic of North Macedonia; BG: Bulgaria.
