# Supplementary material for: Persistent Moderate-to-Weak Mediterranean Diet Adherence and Low Scoring for Plant-Based Foods across Several Southern European Countries: Are We Overlooking the Mediterranean Diet Recommendations?
Source: Nutrients. 2021 Apr 23;13(5):1432. doi: 10.3390/nu13051432 (PMC8145023; doi:10.3390/nu13051432)
Supplement: Supplementary file 1 [file nutrients-13-01432-s001.zip › Supplementary Table S1.pdf]

Supplementary Table S1.- Summary of reported diseases per country as classified by the International Statistical Classification of Diseases and Related Health Problems (ICD, WHO). SP: Spain; IT: Italy; PT: Portugal; GR: Greece; CY: Cyprus; BG: Bulgaria; NMK: Republic of North Macedonia; MED: all Mediterranean countries together; Non-Med: all non-Mediterranean countries together.

ICD	SP	IT	PT	GR	CY	MED	BG	NMK	Non-MED
1. Certain infectious or parasitic diseases	1 (0.5%)[1]	1 (1.1%)	1 (0.8%)	1 (1.2)	-	4 (0.6%)	-	1 (1.5%)	1 (0.8%)
2. Neoplasms	15 (7.6%)	5 (5.3%)	2 (1.6%)	3 (3.5)	2 (1.5%)	27 (4.2%)	3 (4.4%)	9 (13.6%)	12 (9.0%)
3. Diseases of the blood or blood-forming organs	2 (1.0%)	2 (2.1%)	2 (1.6%)	1 (1.2)	2 (1.5%)	9 (1.4%)	2 (3.0%)	2 (3.4%)	4 (3.0%)
4. Diseases of the immune system	10 (5.1%)	7 (7.4%)	8 (6.5%)	2 (2.3)	-	27 (4.2%)	1 (1.5%)	2 (3.4%)	3 (2.2%)
5. Endocrine, nutritional or metabolic diseases	61 (31%)	22 (23.4%)	22 (17.9%)	37 (43.1)	43 (31.6%)	185 (29.1%)	25 (37.3%)	22 (33.4%)	47 (35.4%)
6. Mental, behavioural or neurodevelopmental disorders	5 (2.5%)	4 (4.3%)	13 (10.7%)	3 (3.5)	2 (1.5%)	27 (4.2%)	2 (3.0%)	1 (1.5%)	3 (2.2%)
7. Sleep-wake disorders	4 (2.0%)	-	-	-	-	4 (0.6%)	1 (1.5%)		1 (0.8%)
8. Diseases of the nervous system	4 (2.0%)	1 (1.1%)	7 (5.7%)	4 (4.6)	5 (3.7%)	21 (3.3%)	1 (1.5%)	2 (3.4%)	3 (2.2%)
9. Diseases of the visual system	4 (2.0%)	-	1 (0.8%)	-	-	5 (0.8%)	-	2 (3.4%)	2 (1.5%)
10. Diseases of the ear or mastoid process	-	-	1 (0.8%)	-	-	1 (0.2%)	-	-	-
11. Diseases of the circulatory system	34 (17.1%)	27 (28.7%)	21 (17.1%)	3 (3.5)	21 (15.4%)	106 (16.7%)	11 (16.4%)	15 (22.8%)	26 (19.5%)
12. Diseases of the respiratory system	12 (6.1%)	2 (2.1 %)	22 (17.9%)	10 (11.6)	1 (0.7%)	47 (7.4%)	1 (1.5%)	3 (3.4%)	4 (3.0%)
13. Diseases of the digestive system	13 (6.6%)	11 (11.7%)	14 (11.4%)	7 (8.1)	12 (8.8%)	57 (8.9%)	12 (18.0%)	3 (3.4%)	15 (11.3%)
14. Diseases of the skin	5 (2.5%)	-	-	2 (2.3)	4 (2.9%)	11 (1.7%)	1 (1.5%)	-	1 (0.8%)
15. Diseases of the musculoskeletal system or connective tissue	18 (9.1%)	5 (5.3%)	1 (0.8%)	9 (10.5)	39 (28.7%)	72 (11.4%)	5 (7.4%)	2 (3.4%)	7 (5.3%)
16. Diseases of the genitourinary system	4 (2.0%)	6 (6.4%)	2 (1.6%)	4 (4.6)	1 (0.7%)	17 (2.7%)	1 (1.5%)	2 (3.4%)	3 (2.2%)
17. Conditions related to sexual health	-	-	-	-	-	-	-	-	-
18. Pregnancy, childbirth or puerperium	-	-	1 (0.8%)	-	1 (0.7%)	2 (0.3%)	-	-	-
19. Certain conditions originating in the perinatal period	-	-	-	-	-	-	-	-	-
20. Developmental anomalies	-	-	-	-	-	-	-	-	-
21. Symptoms, signs or clinical findings, not elsewhere classified	6 (3.0%)	1 (1.1%)	5 (4.1%)	-	3 (2.2%)	15 (2.3%)	1 (1.5%)	-	1 (0.8%)
Total reported	198	94	123	86	136	637	67	66	133

[1]: Results are shown as number of cases reported (% of total).
