# Supplementary material for: Persistent Moderate-to-Weak Mediterranean Diet Adherence and Low Scoring for Plant-Based Foods across Several Southern European Countries: Are We Overlooking the Mediterranean Diet Recommendations?
Source: Nutrients. 2021 Apr 23;13(5):1432. doi: 10.3390/nu13051432 (PMC8145023; doi:10.3390/nu13051432)
Supplement: Supplementary file 1 [file nutrients-13-01432-s001.zip › Supplementary Table S2a.- 14MEDAS_MEDcountries_final.pdf]

Supplementary Table S2a. 14-MEDAS scoring throughout the MED sample population: score values are presented as the median (IQR, interquartile range), the mean ± SD and the distribution (*N* (%) into low (≤ 5), moderate (6 – 9) and high (≥ 10) levels of MD adherence. SP: Spain; IT: Italy; PT: Portugal; GR: Greece; CY: Cyprus; MED: All Mediterranean countries together.

Country (*N*)	MED (2163)	SP (467)	IT (503)	PT (488)	GR (288)	CY (417)	*p*-value
Median (IQR)	7.00 (2.00)	8.00 (2.00)	7.00 (2.00)	7.00 (3.00)	7.00 (3.00)	6.00 (2.00)	
Mean ± SD	7.08 ± 1.96	7.90 ± 1.72	6.80 ± 1.54	7.38 ± 2.10	7.13 ± 2.03	6.12 ± 1.99	<0.001*
Low	448 (20.7)	33 (7.1)	99 (19.7)	97 (19.9)	65 (22.6)	154 (36.9)	
Moderate	1477 (68.3)	355 (76.0)	384 (76.3)	306 (62.7)	189 (65.6)	243 (58.3)	<0.001
High	238 (11.0)	79 (16.9)	20 (4.0)	85 (17.4)	34 (11.8)	20 (4.8)	
Sex							
Women	7.00 (3.00)	8.00 (2.00)	7.00 (2.00)	8.00 (3.00)	7.00 (3.00)	6.00 (2.00)	
	7.22 ± 1.92	8.05 ± 1.65	6.89 ± 1.59	7.71 ± 1.96	7.23 ± 2.05	6.22 ± 1.82	<0.001
Low	274 (19.0)	15 (5.6)	59 (18.8)	50 (14.3)	52 (23.3)	97 (32.9)	
Moderate	995 (68.8)	202 (75.9)	240 (76.6)	229 (65.6)	141 (63.2)	184 (62.4)	<0.001
High	177 (12.2)	49 (18.4)	14 (4.6)	70 (20.1)	30 (13.5)	14 (4.7)	
Men	7.00 (2.00)	8.00 (3.00)	7.00 (2)	7.00 (3.00)	7.00 (2.00)	6.00 (4.00)	
	6.81 ± 2.01	7.71 ± 1.80	6.64 ± 1.43	6.58 ± 2.25	6.77 ± 1.92	5.88 ± 2.31	<0.001
Low	172 (24.1)	18 (9.0)	40 (21.0)	46 (33.8)	13 (20.0)	56 (46.7)	
Moderate	479 (67.3)	153 (76.1)	144 (75.8)	75 (55.1)	48 (73.8)	58 (48.3)	<0.001
High	61 (8.6)	30 (14.9)	6 (3.2)	15 (11.0)	4 (6.2)	6 (5.0)	
p-value (M-W)	<0.001	0.029	0.067	<0.001	0.104	0.076	
p-value (Chi-S)	0.002	0.271	0.664	<0.001	0.184	0.026	
Age range							
18–24 y	7.00 (3.00)	8.00 (3.00)	6.00 (2.00)	7.00 (3.00)	7.00 (4.00)	6.00 (2.00)	
	6.53 ± 1.88	7.43 ± 1.86	6.26 ± 1.52	6.71 ± 2.09	6.48 ± 2.1	6.04 ± 1.78	0.001
Low	106 (28.9)	8 (18.2)	34 (28.8)	37 (29.8)	8 (32.0)	18 (32.7)	
Moderate	238 (64.8)	31 (70.5)	81 (68.6)	76 (61.3)	15 (60.0)	35 (63.6)	0.305
High	23 (6.3)	5 (11.4)	3 (2.6)	11 (8.9)	2 (8.0)	2 (3.6)	
25–44 y	7.00 (2.00)	7.50 (2.0)	7.0 (2.0)	8.00 (3.00)	7.00 (3.00)	6.00 (2.00)	
	6.91 ± 1.92	7.58 ± 1.49	6.75 ± 1.49	7.50 ± 2.03	7.08 ± 1.97	6.00 ± 1.94	<0.001
Low	240 (23.0)	11 (7.9)	44 (20.5)	38 (17.7)	51 (22.8)	97 (38.6)	
Moderate	712 (68.2)	114 (81.4)	164 (76.3)	138 (64.2)	151 (67.4)	145 (57.8)	<0.001
High	92 (8.8)	15 (10.7)	7 (3.2)	39 (18.1)	22 (9.8)	9 (3.6)	
45–64 y	7.00 (3.00)	8.00 (2.00)	7.00 (2.00)	8.00 (3.50)	8.00 (4.00)	6.00 (3.00)	
	7.56 ± 1.96	8.10 ± 1.78	7.13 ± 1.45	7.76 ± 2.16	7.91 ± 2.21	6.34 ± 2.10	<0.001
Low	98 (14.1)	14 (5.2)	20 (13.4)	22 (15.6)	5 (14.3)	37 (37.0)	
Moderate	481 (69.3)	198 (73.6)	124 (83.2)	84 (59.6)	20 (57.1)	55 (55.0)	<0.001
High	115 (16.6)	57 (21.3)	5 (3.5)	35 (24.8)	10 (28.6)	8 (8.0)	
≥ 65 y	8.00 (2.00)	9.00 (1.00)	8.00 (4.0)	8.00 (2.50)	7.00	7.00 (3.00)	
	7.89 ± 1.82	8.69 ± 1.49	7.86 ± 1.68	7.88 ± 1.25	7.00	7.09 ± 2.59	0.543
Low	3 (5.5)	0 (0.0)	1 (4.8)	0 (0.00)	0 (0)	2 (18.2)	
Moderate	44 (81.5)	12 (92.3)	15 (71.4)	8 (100.0)	1 (100)	8 (72.7)	0.359
High	7 (13.0)	1 (7.7)	5 (23.8)	0 (0.0)	0 (0)	1 (9.1)	
p-value (K-W)	<0.001	0.010	<0.001	<0.001	0.074	0.218	
p-value (Chi-S)	<0.001	<0.001	<0.001	0.001	0.052	0.440	
ρ (*p*-value)	0.21 (<0.001)	0.15 (0.002)	0.29 (<0.001)	0.18 (<0.001)	0.15 (0.016)	0.11 (0.025)	
BMI range							
< 18.5 Kg/m^2	7.00 (3.00)	7.00 (2.8)	7.00 (2.25)	8.00 (1.50)	7.00 (4.00)	7.00 (3.00)	
	6.84 ± 1.85	6.89 ± 1.96	7.13 ± 1.88	7.77 ± 1.52	7.33 ± 2.39	6.44 ± 1.65	0.090
Low	20 (27.0)	2 (25.0)	3 (13.6)	2 (11.8)	2 (22.2)	6 (33.3)	
Moderate	48 (64.9)	5 (62.5)	17 (77.3)	13 (76.5)	5 (55.6)	12 (66.7)	0.472
High	6 (8.1)	1 (12.5)	2 (9.1)	2 (11.8)	2 (22.2)	0 (0.0)	
18.5 - 24.9 Kg/m^2	7.00 (3.00)	8.00 (2.00)	7.00 (2.00)	7.00 (3.00)	8.00 (3.00)	6.00 (3.00)	
	7.19 ± 1.97	8.06 ± 1.71	6.82 ± 1.43	7.42 ± 2.25	7.39 ± 2.04	6.27 ± 1.91	<0.001
Low	237 (19.0)	15 (5.6)	52 (17.3)	65 (22.0)	27 (19.0)	75 (31.4)	
Moderate	854 (68.3)	201 (75.3)	239 (79.7)	167 (56.4)	95 (66.9)	152 (63.6)	<0.001

High	159 (12.7)	51 (19.1)	9 (3.0)	64 (21.6)	20 (14.1)	12 (5.0)	
25.0 - 29.9 Kg/m²	7.00 (2.00)	8.00 (3.00)	7.00 (3.00)	7.00 (3.00)	7.00 (3.00)	6.00 (3.00)	
	7.06 ± 1.97	7.73 ± 1.80	6.65 ± 1.58	7.36 ± 1.79	6.87 ± 2.06	6.04 ± 2.31	<0.001
Low	119 (21.1)	14 (9.8)	35 (24.8)	19 (17.4)	22 (26.5)	40 (42.1)	
Moderate	390 (69.3)	106 (74.1)	101 (71.6)	79 (72.5)	54 (65.1)	49 (51.6)	<0.001
High	54 (9.6)	23 (16.1)	5 (3.6)	11 (10.1)	7 (8.4)	6 (6.3)	
≥ 30 Kg/m²	7.00 (3.00)	8.00 (2.00)	7.00 (2.00)	7.00 (2.00)	7.00 (3.00)	5.00 (3.00)	
	6.66 ± 1.91	7.82 ± 1.45	6.84 ± 1.80	7.14 ± 2.23	6.73 ± 1.73	5.59 ± 1.77	<0.001
Low	69 (27.8)	2 (4.5)	8 (21.0)	10 (19.2)	13 (25.5)	32 (50.8)	
Moderate	162 (65.3)	38 (86.4)	27 (71.0)	35 (67.3)	34 (66.7)	29 (46.0)	<0.001
High	17 (6.9)	4 (9.1)	3 (8.0)	7 (13.5)	4 (7.8)	2 (3.2)	
p-value (K-W)	0.001	0.142	0.045	0.749	0.073	0.030	
p-value (Chi-S)	0.005	0.142	0.135	0.048	0.580	0.076	
ρ (*p*-value)	-0.11 (<0.001)	-0.08 (0.089)	-0.05 (0.257)	-0.09 (0.051)	-0.18 (0.003)	-0.17 (0.001)	
Disease status							
No pathology	7.00 (2.00)	8.00 (2.00)	7.00 (2.00)	7.00 (3.00)	7.00 (3.00)	6.00 (2.00)	
	7.02 ± 1.96	7.86 ± 1.79	6.79 ± 1.50	7.34 ± 2.15	7.04 ± 2.06	6.15 ± 1.99	<0.001
Low	338 (21.2)	26 (8.6)	80 (19.0)	71 (20.8)	50 (23.1)	111 (35.1)	
Moderate	1090 (68.2)	223 (73.8)	327 (77.5)	208 (60.8)	144 (66.7)	188 (59.5)	<0.001
High	170 (10.6)	53 (17.5)	15 (3.5)	63 (18.4)	22 (10.2)	17 (5.4)	
One pathology	7.00 (3.00)	8.00 (2.00)	7.00 (2.00)	8.00 (3.00)	7.00 (3.00)	6.00 (4.00)	
	7.27 ± 1.95	7.88 ± 1.61	6.83 ± 1.78	7.58 ± 1.91	7.47 ± 1.89)	5.99 ± 2.14	<0.001
Low	79 (18.5)	5 (4.1)	16 (22.8)	17 (16.0)	11 (17.7)	31 (44.3)	
Moderate	298 (69.6)	99 (81.8)	49 (70.0)	73 (68.9)	40 (64.6)	37 (52.9)	<0.001
High	51 (11.9)	17 (14.0)	5 (7.2)	16 (15.1)	11 (17.7)	2 (2.9)	
Multi-pathology	7.00 (3.00)	8.50 (2.00)	7.00 (3.00)	8.50 (2.50)	7.00 (4.00)	6.00 (2.00)	
	7.29 ± 1.89	8.22 ± 1.41	6.91 ± 1.58	8.20 ± 2.30	7.22 ± 2.10	6.13 ± 1.59	<0.001
Low	21 (21.4)	1 (2.8)	3 (27.3)	1 (10.0)	3 (33.3)	12 (38.7)	
Moderate	66 (67.3)	29 (80.6)	8 (72.7)	6 (60.0)	5 (55.6)	18 (58.1)	0.006
High	11 (11.2)	6 (16.7)	0 (0)	3 (30.0)	1 (11.1)	1 (3.2)	
p-value (K-W)	0.022	0.390	0.970	0.205	0.362	0.856	
p-value (Chi-S)	0.763	0.296	0.456	0.466	0.456	0.610	

N: sample population size. The Mann–Whitney test (M-W) was used to assess the differences in the 14-MEDAS score between sexes. The Kruskal-Wallis (K-W) test was applied for comparisons of the 14-MEDAS score across age ranges, BMI categories and disease status subgroups. Chi-Square tests (Chi-S) were used to assess differences between nominal and ordinal variables. Correlation between variables was calculated using the Spearman coefficient (ρ) adjusted in each case for the corresponding confounding variables (sex, age, BMI, disease). *: *p*-value adjusted for sex, age, BMI and disease (General Linear Model); differences were considered significant when *p*-values < 0.05.
