# Supplementary material for: Persistent Moderate-to-Weak Mediterranean Diet Adherence and Low Scoring for Plant-Based Foods across Several Southern European Countries: Are We Overlooking the Mediterranean Diet Recommendations?
Source: Nutrients. 2021 Apr 23;13(5):1432. doi: 10.3390/nu13051432 (PMC8145023; doi:10.3390/nu13051432)
Supplement: Supplementary file 1 [file nutrients-13-01432-s001.zip › Supplementary Table S2b.- 14MEDAS_Non-MEDcountries_final.pdf]

Supplementary Table S2b. 14-MEDAS scoring throughout the non-Mediterranean sample population: score values are presented as the median (IQR, interquartile range), the mean ± SD and the distribution (*N* (%) into low (≤ 5), moderate (6 – 9) and high (≥ 10) levels of MD adherence. BG: Bulgaria; NMK: Republic of North Macedonia; non-MED: all non-Mediterranean countries together.

Country (*N*)	Non-MED (783)	BG (440)	NMK (343)	*p*-value
Median (IQR)	5.00 (3.00)	6.00 (2.00)	5.00 (3.00)	
Mean ± SD	5.58 ± 1.82	5.80 ± 1.80	5.30 ± 1.80	<0.001*
Low	402 (51.3)	199 (45.2)	203 (59.2)	
Moderate	363 (46.4)	230 (52.3)	133 (38.8)	<0.001
High	18 (2.3)	11 (2.5)	7 (2.0)	
Sex				
Women	6.00 (3.00)	6.00 (2.00)	5.00 (3.00)	
	5.66 ± 1.85	5.9 ± 1.80	5.30 ± 1.90	<0.001
Low	276 (48.8)	126 (41.6)	150 (57.0)	
Moderate	277 (48.9)	169 (55.8)	108 (41.1)	<0.001
High	13 (2.3)	8 (2.6)	5 (1.9)	
Men	5.00 (2.00)	5.00 (1.00)	5.00 (2.00)	
	5.37 ± 1.73	5.52 ± 1.70	5.11 ± 1.75	0.058
Low	126 (58.1)	73 (53.3)	53 (66.3)	
Moderate	86 (39.6)	61 (44.5)	25 (31.3)	0.155
High	5 (2.3)	3 (2.2)	2 (2.5)	
p-value (M-W)	0.034	0.017	0.281	
p-value (Chi-S)	0.062	0.074	0.285	
Age range				
18–24 y	5.00 (2.00)	5.00 (3.00)	5.00 (20)	
	4.83 ± 1.78	5.18 ± 1.68	4.78 ± 1.88	0.359
Low	127 (70.2)	15 (68.2)	112 (70.4)	
Moderate	52 (28.7)	7 (31.8)	45 (28.3)	0.829
High	2 (1.1)	0 (0.00)	2 (1.3)	
25–44 y	6.00 (2.75)	6.00 (2.00)	5.00 (3)	
	5.72 ± 1.83	5.76 ± 1.83	5.64 ± 1.84	0.424
Low	166 (46.1)	99 (43.2)	67 (51.1)	
Moderate	183 (50.8)	123 (53.7)	60 (45.8)	0.340
High	11 (3.1)	7 (3.1)	4 (3.1)	
45–64 y	6.00 (2.00)	6.00 (2.00)	6.00 (200)	
	6.02 ± 1.72	6.02 ± 1.78	6.02 ± 1.65	0.984
Low	93 (42.9)	70 (41.9)	23 (46.0)	
Moderate	119 (54.8)	93 (55.7)	26 (52.0)	0.873
High	5 (2.3)	4 (2.4)	1 (2.0)	
≥ 65 y	5.00 (1.50)	5.00 (2.00)	6.00 (1.0)	
	5.28 ± 1.13	5.18 ± 1.14	6.00 ± 1.0	0.222
Low	16 (64.0)	15 (68.2)	1 (33.34)	
Moderate	9 (36.0)	7 (31.8)	2 (66.7)	0.238
High	0 (0.)	0 (0)	0 (0)	
p-value (K-W)	<0.001	0.021	<0.001	
p-value (Chi-S)	<0.001	0.085	0.010	
ρ (p-value)	0.19 (<0.001)	0.03 (0.526)	0.25 (<0.001)	
BMI range				
< 18.5 Kg/m^2	5.00 (2.00)	6.00 (4.00)	4.50 (3.00)	
	5.32 ± 2.32	5.96 ± 2.20	4.50 ± 2.28	0.041
Low	23 (56.1)	11 (47.8)	12 (66.7)	
Moderate	16 (39.0)	11 (47.8)	5 (27.8)	0.425
High	2 (4.9)	1 (4.4)	1 (5.6)	
18.5 - 24.9 Kg/m^2	5.00 (3.00)	6.00 (2.00)	5.00 (2.00)	
	5.60 ± 1.78	5.89 ± 1.66	5.27 ± 1.85	<0.001
Low	226 (51.8)	100 (43.5)	126 (61.2)	
Moderate	199 (45.6)	124 (53.9)	75 (36.4)	0.001

High	11 (2.5)	6 (2.6)	5 (2.4)	
25.0 - 29.9 Kg/m^2	5.00 (2.00)	6.00 (2.00)	5.00 (2.00)	
	5.69 ± 1.78	5.76 ± 1.91	5.60 ± 1.61	0.568
Low	101 (51.3)	54 (49.1)	47 (54.0)	
Moderate	93 (47.2)	53 (48.2)	40 (46.0)	0.265
High	3 (1.5)	3 (2.7)	0 (0)	
≥ 30 Kg/m^2	6.00 (3.00)	6.00 (3.00)	5.00 (4.00)	
	5.49 ± 1.81	5.53 ± 1.75	5.33 ± 2.08	0.466
Low	46 (46.9)	34 (44.2)	12 (57.1)	
Moderate	50 (51.0)	42 (54.5)	8 (38.1)	0.296
High	2 (2.0)	1 (1.3)	1 (4.8)	
p-value (K-W)	0.450	0.680	0.065	
p-value (Chi-S)	0.760	0.920	0.381	
ρ (*p*-value)	-0.01 (0.876)	-0.07 (0.172)	0.10 (0.078)	
Disease status				
No pathology	5.00 (3.00)	6.00 (2.00)	5.00 (2.00)	
	5.54 ± 1.80	5.75 ± 1.73	5.26 ± 1.86	<0.001
Low	341 (52.7)	169 (46.5)	172 (60.6)	
Moderate	292 (45.1)	187 (51.5)	105 (37.0)	0.001
High	14 (2.2)	7 (2.0)	7 (2.5)	
One pathology	6.00 (2.00)	6.00 (3.00)	6.00 (3.00)	
	5.95 ± 1.90	6.19 ± 1.93	5.67 ± 1.85	0.236
Low	42 (42.5)	20 (37.0)	22 (48.9)	
Moderate	54 (54.5)	31 (57.4)	23 (51.1)	0.175
High	3 (3.0)	3 (5.6)	0.0 (0.0)	
Multi-pathology	6.00 (2.75)	6.00 (5.00)	5.50 (2.50)	
	5.81 ± 2.31	6.00 ± 2.74	5.50 ± 1.51	0.826
Low	7 (43.8)	4 (40.0)	3 (50.0)	
Moderate	8 (50.0)	5 (50.0)	3 (50.0)	0.710
High	1 (6.3)	1 (10.0)	0.0 (0.0)	
p-value (K-W)	0.117	0.354	0.271	
p-value (Chi-S)	0.292	0.205	0.353	

N: sample population size. The Mann–Whitney test (M-W) was used to assess the differences in the 14-MEDAS score between sexes. The Kruskal-Wallis (K-W) test was applied for comparisons of the 14-MEDAS score across age ranges, BMI categories and disease status subgroups. Chi-Square tests (Chi-S) were used to assess differences between nominal and ordinal variables. Correlation between variables was calculated using the Spearman coefficient (ρ) adjusted in each case for the corresponding confounding variables (sex, age, BMI, disease).; *: *p*-value across countries adjusted for sex, age, BMI and disease (General Linear Model); differences were considered significant when *p*-values < 0.05.
