# Supplementary material for: Persistent Moderate-to-Weak Mediterranean Diet Adherence and Low Scoring for Plant-Based Foods across Several Southern European Countries: Are We Overlooking the Mediterranean Diet Recommendations?
Source: Nutrients. 2021 Apr 23;13(5):1432. doi: 10.3390/nu13051432 (PMC8145023; doi:10.3390/nu13051432)
Supplement: Supplementary file 1 [file nutrients-13-01432-s001.zip › Supplementary Table S3.- MED vs nonMED and SEX_final.pdf]

Supplementary Table S3. Food choices differences between women and men in the sample population from the MED and the non-MED group of countries: % of participants scoring 1 (within Mediterranean diet recommendations) for each question of the 14-MEDAS. MED: all Mediterranean countries together (SP, PT, IT, GR, CY); Non-MED: all non-Mediterranean countries together (BG and NMK).

	MED				Non-MED			
14-MEDAS Question (Score 1)	Women	Men	*p*-value[1]	Effect size[2]	Women	Men	*p*-value[1]	Effect size[2]
1. Olive oil as main culinary fat (yes)	95.4	93.2	0.03	0.05 (small)	38.7	36.3	NS	-
2. Olive oil (≥4 tbsp[3]/day)	18.4	15.5	NS	-	6.9	5.2	NS	-
3. Vegetables (≥2 portions/day)	57.3	39.6	<0.001	0.17 (small)	34.2	30.4	NS	-
4. Fruits (≥3 portions/day)	19.3	17.7	NS	-	8.5	8.9	NS	-
5. Red meat (<1 portion/day)	98.9	97.5	0.018	0.05 (small)	98.3	91.8	<0.001	0.16 (small)
6. Butter or cream (<1 portion/day)	82.9	81.5	NS	-	70.5	75.1	NS	-
7. Sweet drinks (<1 drink/day)	80.1	73.4	<0.001	0.08 (small)	78.9	74.1	NS	-
8. Wine (7 to 14 glasses/week)	2.3	6.4	<0.001	0.10 (small)	8.5	8.9	NS	-
9. Legumes (≥3 portions/week)	15.6	22.4	<0.001	0.08 (small)	11.2	8.9	NS	-
10. Fish/seafood (≥3 portions/week)	15.9	12.6	0.04	0.04 (small)	2.2	3.2	NS	-
11. Desserts (<3 portions/week)	76.3	76.8	NS	-	67.0	73.0	NS	-
12. Nuts (≥3 portions/week)	28.9	25.5	NS	-	16.0	13.3	NS	-
13. White meat preference (yes)	82.1	67.8	<0.001	0.16 (small)	56.9	45.7	0.002	0.10 (small)
14. 'Sofrito'[4] (≥2 meals/week)	59.5	64.4	0.03	0.05 (small)	60.2	47.8	0.001	0.11 (small)

[1] Comparison between sexes: Chi-Square *p*-value; [2] Strength of association or effect size by Phi and Cramer's *V* association coefficients (small 0.1, median 0.3, large 0.5); [3] tbsp: table spoon; [4] 'Sofrito': mixture of tomato, onion and (or) garlic fried in olive oil use for cooking meals; NS: Not significant. SP: Spain; PT: Portugal; IT: Italy; GR: Greece; CY: Cyprus; BG: Bulgaria; NMK: Republic of North Macedonia.
