# Supplementary material for: Persistent Moderate-to-Weak Mediterranean Diet Adherence and Low Scoring for Plant-Based Foods across Several Southern European Countries: Are We Overlooking the Mediterranean Diet Recommendations?
Source: Nutrients. 2021 Apr 23;13(5):1432. doi: 10.3390/nu13051432 (PMC8145023; doi:10.3390/nu13051432)
Supplement: Supplementary file 1 [file nutrients-13-01432-s001.zip › Supplementary Table S4.- MED vs nonMED and AGE range_final.pdf]

Supplementary Table S4. Food choices differences across age ranges in the sample population from the MED and the non-MED group of countries: % of participants scoring 1 (within Mediterranean diet recommendations) for each question of the 14-MEDAS. MED: all Mediterranean countries together (SP, PT, IT, GR, CY); Non-MED: all non-Mediterranean countries together (BG and NMK).

	MED						Non-MED					
14-MEDAS Question (Score 1)	18-24 y	25-44 y	45-64 y	≥ 65 y	*p*-value[1]	Effect size[2]	18-24 y	25-44 y	45-64 y	≥ 65 y	*p*-value[1]	Effect size[2]
1. Olive oil as main culinary fat (yes)	90.7	94.4	96.8	98.2	<0.001	0.09 (small)	26.8	43.9	41.3	28.6	<0.001	0.15 (small)
2. Olive oil (≥4 tbsp[3]/day)	11.1	17.9	19.8	15.8	0.004	0.08 (small)	2.9	9.5	5.3	0.0	0.006	0.12 (small)
3. Vegetables (≥2 portions/day)	45.2	53.4	51.3	58.6	0.03	0.06 (small)	21.0	30.5	52.2	21.4	<0.001	0.25 (small)
4. Fruits (≥3 portions/day)	10.9	16.2	25.4	34.5	<0.001	0.15 (small)	6.5	8.8	10.9	7.1	NS	-
5. Red meat (<1 portion/day)	96.8	98.6	99.0	98.2	0.046	0.06 (small)	95.9	96.2	97.4	96.4	NS	-
6. Butter or cream (<1 portion/day)	81.2	81.5	83.8	87.7	NS	-	73.3	70.9	71.3	74.1	NS	-
7. Sweet drinks (<1 drink/day)	68.4	76.7	84.1	83.9	<0.001	0.13 (small)	61.3	79.4	89.5	88.9	<0.001	0.25 (small)
8. Wine (7 to 14 glasses/week)	2.4	2.2	5.9	10.5	<0.001	0.11 (small)	2.5	8.0	15.7	10.7	<0.001	0.17 (small)
9. Legumes (≥3 portions/week)	15.9	15.4	22.0	22.4	0.002	0.08 (small)	11.5	11.6	7.8	7.1	NS	-
10. Fish/seafood (≥3 portions/week)	15.1	11.9	18.9	15.5	0.001	0.09 (small)	1.7	2.5	3.5	0.0	NS	-
11. Desserts (<3 portions/week)	71.6	75.1	79.5	94.7	<0.001	0.10 (small)	51.7	69.3	83.0	92.9	<0.001	0.26 (small)
12. Nuts (≥3 portions/week)	15.1	28.7	32.5	35.1	<0.001	0.14 (small)	13.2	17.4	15.2	3.6	NS	-
13. White meat preference (yes)	77.3	76.3	79.2	73.2	NS	-	58.6	57.4	44.8	35.7	0.001	0.13 (small)
14. 'Sofrito'[4] (≥2 meals/week)	61.2	55.4	68.8	70.7	<0.001	0.13 (small)	48.1	60.2	60.4	50.0	0.012	0.11 (small)

[1] Comparison between age ranges: Chi-Square *p*-value; [2] Strength of association or effect size by Phi and Cramer's *V* association coefficients (small 0.1, median 0.3, large 0.5); [3] tbsp: table spoon; [4] 'Sofrito': mixture of tomato, onion and (or) garlic fried in olive oil use for cooking meals; NS: Not significant. SP: Spain; PT: Portugal; IT: Italy; GR: Greece; CY: Cyprus; BG: Bulgaria; NMK: Republic of North Macedonia.
