# Supplementary material for: Persistent Moderate-to-Weak Mediterranean Diet Adherence and Low Scoring for Plant-Based Foods across Several Southern European Countries: Are We Overlooking the Mediterranean Diet Recommendations?
Source: Nutrients. 2021 Apr 23;13(5):1432. doi: 10.3390/nu13051432 (PMC8145023; doi:10.3390/nu13051432)
Supplement: Supplementary file 1 [file nutrients-13-01432-s001.zip › Supplementary Table S5.- MED vs nonMED and BMI range_final.pdf]

Supplementary Table S5. Food choices differences across BMI ranges (kg/m^2) in the sample population from the MED and the non-MED group of countries: % of participants scoring 1 (within Mediterranean diet recommendations) for each question of the 14-MEDAS. MED: all Mediterranean countries together (SP, PT, IT, GR, CY); Non-MED: all non-Mediterranean countries together (BG and NMK).

	MED						Non-MED					
14-MEDAS Question (Score 1)	< 18.5	18.5 - 24.9	25.0 - 29.9	≥ 30	*p*-value[1]	Effect size[2]	< 18.5	18.5 - 24.9	25.0 - 29.9	≥ 30	*p*-value[1]	Effect size[2]
1. Olive oil as main culinary fat (yes)	92.3	94.7	96.4	91.3	0.021	0.07 (small)	45.5	37.0	41.3	33.9	NS	-
2. Olive oil (≥4 tbsp[3]/day)	17.9	16.9	18.6	17.7	NS	-	14.6	7.0	4.2	5.8	NS	-
3. Vegetables (≥2 portions/day)	44.9	54.3	49.1	45.9	0.020	0.07 (small)	25.0	31.7	36.2	37.6	NS	-
4. Fruits (≥3 portions/day)	12.8	19.0	19.4	16.9	NS	-	4.5	9.5	8.7	7.3	NS	-
5. Red meat (<1 portion/day)	98.7	98.4	98.8	98.4	NS	-	97.7	98.0	96.1	91.0	0.003	0.12 (small)
6. Butter or cream (<1 portion/day)	84.4	83.6	81.3	78.4	NS	-	65.9	70.8	77.6	66.1	NS	-
7. Sweet drinks (<1 drink/day)	72.7	78.5	80.3	70.9	0.014	0.07 (small)	59.1	77.4	80.1	81.1	0.016	0.11 (small)
8. Wine (7 to 14 glasses/week)	3.9	3.2	5.1	2.0	NS	-	4.5	9.1	7.1	11.2	NS	-
9. Legumes (≥3 portions/week)	14.1	18.0	18.0	17.7	NS	-	11.4	10.8	11.0	9.0	NS	-
10. Fish/seafood (≥3 portions/week)	14.1	15.4	12.3	16.5	NS	-	4.5	2.0	2.6	2.8	NS	-
11. Desserts (<3 portions/week)	72.7	77.8	74.3	76.9	NS	-	70.5	68.9	67.4	72,1	NS	-
12. Nuts (≥3 portions/week)	22.1	27.9	28.8	26.3	NS	-	11.4	14.9	16.7	14.4	NS	-
13. White meat preference (yes)	80.5	80.3	73.6	70.4	<0.001	0.09 (small)	54.5	56.6	51.9	43.8	NS	-
14. 'Sofrito'[4] (≥2 meals/week)	68.8	60.8	63.5	54.1	0.035	0.06 (small)	47.7	58.3	55.9	57.1	NS	-

[1] Comparison between body mass index (BMI) ranges: Chi-Square *p*-value; [2] Strength of association or effect size by Phi and Cramer's *V* association coefficients (small 0.1, median 0.3, large 0.5); [3] tbsp: table spoon; [4] 'Sofrito': mixture of tomato, onion and (or) garlic fried in olive oil use for cooking meals; NS: Not significant. SP: Spain; PT: Portugal; IT: Italy; GR: Greece; CY: Cyprus; BG: Bulgaria; NMK: Republic of North Macedonia.
