# Supplementary material for: Persistent Moderate-to-Weak Mediterranean Diet Adherence and Low Scoring for Plant-Based Foods across Several Southern European Countries: Are We Overlooking the Mediterranean Diet Recommendations?
Source: Nutrients. 2021 Apr 23;13(5):1432. doi: 10.3390/nu13051432 (PMC8145023; doi:10.3390/nu13051432)
Supplement: Supplementary file 1 [file nutrients-13-01432-s001.zip › Supplementary Table S6.- MED vs nonMED and DISEASE_final.pdf]

Supplementary Table S6. Food choice differences across reported disease status in the sample population from the MED and the non-MED group of countries: % of participants scoring 1 (within Mediterranean diet recommendations) for each question of the 14-MEDAS. MED: all Mediterranean countries together (SP, PT, IT, GR, CY); Non-MED: all non-Mediterranean countries together (BG and NMK).

	MED					Non-MED				
14-MEDAS Question (Score 1)	No pathology	One pathology	Multi-pathology	*p*-value[1]	Effect size[2]	No pathology	One pathology	Multi-pathology	*p*-value[1]	Effect size[2]
1. Olive oil as main culinary fat (yes)	94.1	96.3	96.0	NS	-	37.9	45.6	22.2	NS	-
2. Olive oil (≥4 tbsp[3]/day)	17.8	15.7	23.0	NS	-	6.6	6.7	5.6	NS	-
3. Vegetables (≥2 portions/day)	50.7	52.8	59.0	NS	-	33.0	32.2	58.8	NS	-
4. Fruits (≥3 portions/day)	17.4	21.8	27.0	0.010	0.07 (small)	8.6	8.8	11.1	NS	-
5. Red meat (<1 portion/day)	98.5	98.4	100.0	NS	-	96.3	96.5	100.0	NS	-
6. Butter or cream (<1 portion/day)	82.4	81.7	84.8	NS	-	71.8	76.5	55.6	NS	-
7. Sweet drinks (<1 drink/day)	77.5	79.1	75.8	NS	-	76.4	86.6	64.7	0.024	0.09 (small)
8. Wine (7 to 14 glasses/week)	4.1	2.5	1.0	NS	-	9.2	7.1	5.6	NS	-
9. Legumes (≥3 portions/week)	16.5	20.6	25.0	0.020	0.06 (small)	10.1	13.2	16.7	NS	-
10. Fish/seafood (≥3 portions/week)	14.6	14.4	17.0	NS	-	2.4	2.7	0.0	NS	-
11. Desserts (<3 portions/week)	76.8	73.5	82.8	NS	-	68.9	63.2	83.3	NS	-
12. Nuts (≥3 portions/week)	26.3	33.4	30.0	0.011	0.07 (small)	13.9	23.5	22.2	0.021	0.09 (small)
13. White meat preference (yes)	76.4	82.7	72.0	0.008	0.07 (small)	53.1	59.6	50.0	NS	-
14. 'Sofrito'[4] (≥2 meals/week)	60.6	63.0	56.0	NS	-	55.5	62.3	72.2	NS	-

[1] Comparison across disease status: Chi-Square *p*-value; [2] Strength of association or effect size by Phi and Cramer's *V* association coefficients (small 0.1, median 0.3, large 0.5); [3] tbsp: table spoon; [4] 'Sofrito': mixture of tomato, onion and (or) garlic fried in olive oil use for cooking meals; NS: Not significant. SP: Spain; PT: Portugal; IT: Italy; GR: Greece; CY: Cyprus; BG: Bulgaria; NMK: Republic of North Macedonia.
